# Lingguizhugan decoction enhances autophagy of Alzheimer’s disease via regulating the mTOR/ p70s6K pathway *in vivo* and *in vitro*

**DOI:** 10.3389/fnagi.2025.1478199

**Published:** 2025-01-22

**Authors:** Xiaojing Chen, Qingrong Tian, Min Gao, Xibin Zhou, Chunxiang Zhou

**Affiliations:** School of Chinese Medicine, Nanjing University of Chinese Medicine, Nanjing, China

**Keywords:** autophagy, Lingguizhugan decoction, mTOR, Chinese medicine pharmacology, neurodegenerative diseases, complementary medicine

## Abstract

**Introduction:**

Lingguizhugan decoction (LGZG) has been reported to treat Alzheimer’s disease (AD) by anti-inflammatory and transporting amyloid-β (Aβ).

**Methods:**

Using APP/PS1 transgenic mice as *in vivo* model and gave LGZG decoction by oral gavage. Using Aβ_25-35_-induced SH-SY5Y cells as *in vitro* model and then added LGZG medicated serum (LMS) to observe the regulatory effect of LGZG on AD autophagy-related pathways. Morris water maze (MWM) was used to evaluate the mice’s learning and memory ability. Mice’s hippocampus tissue sections were stained immunohistochemically to observe hippocampal Aβ deposition. Transmission electron microscopy monitored autophagosomes and autolysosomes. Western blot analysis measured protein expression levels of beclin-1, p62 and light chain 3II (LC3 II) and mTOR signaling. Results: LGZG could greatly improve learning and memory ability of APP/PS1 mice, and enhance autophagy *in vitro* and *in vivo*. LGZG increased the levels of beclin-1 and LC3 II and decreased the levels of p62.

**Conclusion:**

LGZG enhanced autophagy and showed therapeutic potential in AD by inhibiting mTOR/p70s6K signaling.

## 1 Introduction

Alzheimer’s disease (AD), clinically featured with progressive cognitive impairment and memory decline, is the most common age-related dementia, whose percentage is about 60 to 70% of all dementia cases (Jiang et al., 2020). The pathogenesis of AD is rather sophisticated and the Amyloid Cascade Hypothesis (ACH) is one of the most well-accepted hypotheses by far. ACH assumes that the abnormal deposition of amyloid β (Aβ) is the core pathological event of AD, which leads to a sequence of chain reactions such as hyperphosphorylated tau protein misfolding to form neurofibrillary tangles (NFTs), neuroinflammation, amyloidosis diseases and so on. The imbalanced Aβ metabolism (more production and less clearance) is the kickoff session which results in the abnormal deposition of Aβ (Mawuenyega et al., 2010). The clearance of Aβ in the central nervous system (CNS) is mainly through the blood–brain barrier (BBB), glymphatic system, and autophagy-lysosome pathway (Tarasoff-Conway et al., 2015). Wherein, the brain Aβ flows out into the peripheral system via the BBB and glymphatic system while autophagy-lysosome mediates Aβ intracellular degradation. Studies have shown that intraneuronal Aβ accumulation precedes the formation of extracellular senile plaques (Cataldo et al., 2004; Takahashi et al., 2017) and interacts with various organelles such as lysosomes and endoplasmic reticulum to further accelerate AD pathology (Penke et al., 2012). Therefore, targeting intraneuronal Aβ degradation may become an effective method for early prevention and treatment of AD.

Autophagy is an essential cellular self-defense mechanism which protects cells from the effects of stress responses such as DNA damage, hypoxic stress, oxidative stress, and endoplasmic reticulum stress (Turkmen, 2017). When cells are exposed to nutrient/energy deprivation, hypoxia, protein accumulation, organelle damage, or pathogen invasion, autophagy is activated. Autophagy is also considered one of the mechanisms for clearing Aβ from neuronal cells (Kizilarslanoğlu and Ülger, 2015). Activating the autophagy inhibition pathway mTOR signaling pathway will promote hyperphosphorylation and accumulation of tau protein (Ballatore et al., 2007). Clinical trials have found a hyperactivation of the mTOR pathway and a reduction in autophagy in AD patients’ brains (Tramutola et al., 2015). Furthermore, a study has proved that the mTOR inhibitor Rapamycin (Rapa) can activate autophagy and reduce amyloid plaques in AD mice’s hippocampus (Zhang et al., 2017). Therefore, the mTOR signaling pathway could be a prospective treatment target for Aβ-induced brain injury.

The autophagy process is regulated by several key proteins, with mammalian target of rapamycin (mTOR) serving as a central regulator of cell growth and metabolism (Gao et al., 2021). Phosphorylated mTOR (p-mTOR) represents its active state. In neuronal cells, decreased p-mTOR level indicates autophagy activation, which helps to clear abnormal aggregated proteins associated with neurodegenerative diseases (Ghavami et al., 2014; Stekic et al., 2022; Wang and Jia, 2023). p70S6K and 4EBP1 are the downstream effectors of the mTOR pathway involved in protein synthesis (Proud, 2007; Laplante and Sabatini, 2009). When mTOR activation is inhibited, the 4EBP1 level increases while the p70S6K level decreases, thereby reducing protein synthesis and alleviating metabolic burden on cells, creating a more favorable environment for autophagy. The autophagy adapter protein p62 binds to marker proteins and directs them to autophagosomes; its reduced levels reflect active autophagy. Beclin-1 is a key factor in autophagosome formation, and its increased expression indicates enhanced autophagy activity, which helps neurons clear toxic proteins. LC3 II serves as a marker for autophagosome maturation, with LC3 I converting to LC3 II as autophagy activity increases (Mizushima and Komatsu, 2011; Wang et al., 2024). In summary, these coordinated changes in protein expression collectively regulate neuronal autophagy, helping to maintain neuronal homeostasis and reduce deleterious protein accumulation associated with neurodegenerative diseases.

Lingguizhugan decoction (LGZG), a distinguished decoction in traditional Chinese medicine, is a classic recipe for treating AD, fluid retention, and inflammatory lesions (Yu et al., 2014). LGZG is composed of four Chinese herbs, including Poria [*Poria cocos (Schw.)* Wolf], Ramulus Cinnamomi [*Cinnamomum cassia* (L.) J. Presl], Rhizoma Atractylodis Macrocephalae (*Atractylodes macrocephala* Koidz.) and Glycyrrhizae Radix et Rhizoma (*Glycyrrhiza uralensis* Fisch.). We checked all the plant names with http://www.theplantlist.org on November 21, 2023. Traditional Chinese medicine believes Poria cocos promotes urination and drains dampness, Ramulus Cinnamomi warms yang and transforms qi, Rhizoma Atractylodis Macrocephalae can warm spleen yang, transform phlegm and dry dampness, and Glycyrrhizae Radix et Rhizoma coordinates the rest of the ingredients in this formula. The formula has the effect of warming and transforming cold fluid retention, strengthening the spleen and removing dampness (Yu et al., 2014; Xiang et al., 2019; Han et al., 2022; Li et al., 2023; Long et al., 2023; Gao et al., 2024; Ye et al., 2024). The main practical components have been identified in LGZG, including pachymic acid, cinnamic acid, atractylenolide III, glycyrrhizic acid, and glycyrrhiza glycoside (Ji et al., 2018; Li et al., 2023; Li et al., 2024). Preliminary studies by our research group have shown that LGZG inhibited the overexpression of inflammatory factors on Aβ_25-35_-induced SH-SY5Y cell line and BV-2 cell line and reduced the levels of Aβ_1-42_ in Aβ induced-AD rats’ brains.(Xi et al., 2012; Hu et al., 2018). These studies show that LGZG has the potential for AD treatment. Meanwhile, a variety of practical components of Poria [*Poria cocos (Schw.)* Wolf], Ramulus Cinnamomi [*Cinnamomum cassia* (L.) J. Presl] and Glycyrrhizae Radix et Rhizoma (*Glycyrrhiza uralensis* Fisch.) have demonstrated the function of inducing autophagy (Yo et al., 2009; Zhao et al., 2016; Yu et al., 2017; Wang et al., 2019).

Therefore, in this study, we utilized transmission electron microscope (TEM) to examine the ultrastructural features of autophagolysosomes in hippocampal neurons of mice. We also used the fluorescent compound Monodansylcadaverine (MDC), which specifically labels autophagic vesicles, to measure the autophagy level in SH-SY5Y cells. Western blot analysis was used to measure the expression levels of autophagy-associated proteins, including p62, Beclin-1, LC3II, p70S6K, and 4EBP1, to explore whether LGZG can treat AD by regulating autophagy in AD mice.

## 2 Materials and methods

### 2.1 Animals

Five-month-old male SPF grade APP/PS1 mice and wild C57BL/6 mice in the same litter, both weighing 26–32 g, were purchased from Pizhou Dongfang Breeding Co., Ltd. with license number SCXK (Su) 2017 0003. 8-week-old SPF grade SD rats, purchased from Hangzhou Medical College with license number SCXK (Zhejiang) 2019–0002, all weighted 220–250 g. Animal use was approved by the Ethics Committee of the Animal Experiment Center of Nanjing University of Traditional Chinese Medicine. Animal ethical code: Nanjing, China; approval No. 202005A007, No. 202004A031. All animals were raised in the SPF-level experimental room of the Animal Center of Nanjing University of Traditional Chinese Medicine, with a breeding environment of 22–26°C room temperature, 40–60% humidity, and alternating light and dark for 12 h. Regular clean-grade feeds were provided. All mice and rats could eat and drink freely and the padding material was replaced regularly. After 7 days of adaptive feeding, forty APP/PS1 mice were randomly divided into 5 groups: disease group (AD), donepezil hydrochloride group (DNP), LGZG low-dose group (LG-L, 1.75 g/kg/d), LGZG medium dose group (LG-M, 3.5 g/kg/d), LGZG high-dose group (LG-H, 7.0 g/kg/d). Eight wild C57BL/6 mice were used as the control group (CTL). The dosage for mice was based on relevant researches and our previous studies (Fan et al., 2024).

### 2.2 Preparation of LGZG for chemical identification

The composition of LGZG is shown in Table 1. All herbs were purchased from Jiangsu Lianshui Pharmaceutical Co., Ltd. (Lianshui, China) and were mixed in the ratio of 4:3:2:2. All herbs were decocted twice with an 8-fold volume of distilled water for 2 h, pressure-filtered, concentrated to 1 g/ml by rotary evaporation and stored at 4°C for further use. High Performance Liquid Chromatography (HPLC) analysis was conducted for the chemical identification of LGZG decoction.

**Table 1:** Composition of Lingguizhugan decoction (LGZG).

Pharmaceutical name	Chinese name	Latin name	Batch number	Weight (g)
Poria	Fuling	*Poria cocos* (Schw.) Wolf	231011	40
Ramulus Cinnamomi	Guizhi	*Cinnamomum cassia* (L.) J. Presl	230721	30
Rhizoma Atractylodis Macrocephalae	Baizhu	*Atractylodes macrocephala* Koidz.	230831	20
Glycyrrhizae Radix et Rhizoma	Gancao	*Glycyrrhiza uralensis* Fisch.	230501	20

The HPLC analysis procedure is as follows: LGZG preparations (5 ml) were added to a 10 ml centrifuge tube and centrifuged at 10,000 rpm for 10 min. Then, the supernatant filtration was taken with a 0.45 μm filter. Sonicated the supernatant for 5 min at 35 kHz and 25°C and transferred the supernatant (1,000 μl) to a 2 ml sample vial. The HPLC assays are executed on Waters HPLC (Waters e2695 and a 2,489 UV/Visible detector). The chromatography was accomplished on a Waters C18 column (250 mm × 4.6 mm, 5 μm) analyzed at 30°C with the mobile phases of acetonitrile (A) and double-distilled water (B). The flow rate is 0.8 ml/min. The elution conditions were determined using the following gradient program: 0–15 min, 5–15% A; 15–35 min, 15–40% A; 35–40 min, 40–55% A; 40–70 min, 55–85% A; 70–80 min, 85–100% A; 80–95 min, 100–5% A. Inject 10 μl of sample into the HPLC system and detected at 210 nm.

### 2.3 Main reagents

SQSTM1/p62 (D1Q5S) rabbit mAb #39749, GAPDH (D16H11) XP® rabbit mAb #5174, mTOR (7C10) rabbit mAb #2983, Phospho-mTOR (Ser2448; D9C2) XP® rabbit mAb #5536, p70S6Kinase (49D7) rabbit mAb #2708, and 4E-BP1 (53H11) rabbit mAb #9644 were all purchased from Cell Signaling Technology, Inc. (Danvers, MA, USA). Anti-beta amyloid 1–42 antibody [mOC98] (ab201061), goat anti-rabbit IgG H&L (HRP; ab205718), and anti-LC3 II antibody (ab48394) were obtained from Abcam. Other chemicals were purchased from Sigma-Aldrich.

### 2.4 Preparation of LGZG-medicated serum (LMS)

The preparation of LMS were based on our previous studies and relevant references (Xi et al., 2012; Hu et al., 2018; Han et al., 2022). After 7 days of adaptive feeding, thirty-two 8-week-old male SPF grade SD rats were randomly divided into 4 groups with 8 rats in each group: blank control group, LGZG 1.2 g/kg group, LGZG 2.4 g/kg group, and LGZG 4.8 g/kg group. The dosage and method were calculated according to the equivalent dose conversion ratio table based on body surface area for humans and animals in the *Pharmacological Experimental Methodology*. The equivalent dose for rats is 1.2 g/kg. The LGZG low, medium, and high drug serum groups were orally administered with 1, 2, and 4 times the equivalent dose for rats, while the blank group was orally administered with the same volume of physiological saline. Giving gastric lavage twice a day for 7 days. After 1.5 h of the last administration, rats were anesthetized with pentobarbital sodium, and collected blood through the abdominal aorta. After 3 h, centrifuged the blood at 3500 rpm at 4°C for 10 min. Then mixed up the upper serum of the same group, and inactivated it in the 56°C water bath for 30 min. Using the 0.22 μm microporous membrane to filter serum twice and then reserved it in −80°C refrigerator for later use.

### 2.5 Cell line and cell culture

The SH-SY5Y cells (Procell CL-0208) were obtained from Procell Life Science and Engineering Co., Ltd. (Wuhan, China). The complete medium was composed of 10% fetal bovine serum (FBS, 164210–500) MEM/F12 (PM151220) and 1% P/S (PB180120). And the cells were cultured under the condition of 5% CO_2_, 37°C.

### 2.6 Establishment of autophagic-defective model

According to references (Gao et al., 2020; Guo et al., 2022; Ji et al., 2024), SH-SY5Y cells were induced by Aβ_25-35_ (S41991, Shanghaiyuanye Bio-Technology Co., Ltd., Shanghai, China) to establish an autophagic-defective model. Western blot analysis was used to measure LC3 II, p62, and Beclin-1 levels in SH-SY5Y cells at each time point after induced by Aβ_25-35_ to determine the modeling time of AD autophagic-deficient cell model *in vitro*.

### 2.7 Cell grouping, poisoning, and treatment

The cells have been divided into 9 groups as follow: control group (10% drug-free serum), disease group (20 μM Aβ_25-35_ + 10% drug-free serum), LG-L group (20 μM Aβ_25-35_ + 10% 1.2 g/kg LMS), LG-L + 3-MA group (20 μM Aβ_25-35_ + 10% 1.2 g/kg LMS + 5 mM 3-MA), LG-M group (20 μM Aβ_25-35_ + 10% 2.4 g/kg LMS), LG-M + 3-MA group (20 μM Aβ_25-35_ + 10% 2.4 g/kg LMS + 5 mM 3-MA), LG-H group (20 μM Aβ_25-35_ + 10% 4.8 g/kg LMS), LG-H + 3-MA group (20 μM Aβ_25-35_ + 10% 4.8 g/kg LMS + 5 mM 3-MA) and Rapa group (20 μM Aβ_25-35_ + 100 nM Rapa +10% drug-free serum). Cells were inoculated into 6-well plates at 5 × 10^5^ cells per well, and the FBS-containing medium was replaced with serum-free medium. 20 μM Aβ_25-35_ was injected into the disease, LG, and Rapa groups, and the mixture was allowed to stand for 36 h. The cells were then supplemented with a final concentration of 10% LMS and drug-free serum. 3-MA was incorporated into the LG-L + 3-MA group, LG-M + 3-MA group, and LG-H + 3-MA group with a final concentration of 5 mM, and Rapa was administered to the Rapa group with a final concentration of 5 mM. Cells were harvested for subsequent experiments after 12 h of continuous cultivation.

### 2.8 Morris water maze test

A circular pool (120 cm × 50 cm) was the Morris water maze apparatus filled with water, a hidden circular platform (10 cm diameter and submerged 2 cm below the water level), and a record system. Mice were trained twice daily for 3 successive days. A video tracking system recorded the entire process and captured the escape latency (the time between entering the water and standing on the platform). After the forum was successfully located, the mice had 10 s of dwell time. In cases where the mice could not find the platform within 60 s, they were physically placed on the platform for 10 s. The escape latency was recorded on the fourth day, and a single probe trial was carried out on the fifth day.

### 2.9 Immunohistochemistry of Aβ_25-35_

4% paraformaldehyde was used to fix brain tissue at 4°C for 24 h, paraffin-embedded, and sectioned (4 μm). Antigen retrieval was performed using a hotfix method on deparaffinized sections, rehydrated, and washed three times with PBS. The sections were then incubated with goat normal serum (#5425, Cell Signaling Technology) for 20 min at 37°C to block nonspecific binding, followed by incubation with Aβ_25-35_ antibody (1:1000) and negative control with PBS overnight at 4°C. The specimen was then incubated for 20 min at 37°C with primary goat anti-rabbit IgG. The color was developed with 3,3-diaminobenzidine. Eventually, the paraffin section was resin-sealed, baked, and photographed.

### 2.10 Transmission electron microscope

We observed pairs of hippocampal slices with transmission electron microscopy to validate whether LGZG promotes autophagy activation. After perfusion, the brains were taken out, and hippocampal slices were used for transmission electron microscopy. The slides were processed with 1% osmium tetroxide for 2 h at 4°C after three rinses with 0.1 mol/L phosphate buffer (PH 7.2). The samples were dehydrated in graded ethanol and acetone. Finally, the segments were inspected and photographed using a JEOL JEM-1400 EX transmission electron microscope. Three randomly selected fields of view per section were photographed at 10,000 × magnification.

### 2.11 Western blot analysis

Western blot was used to detect the expression of autophagy markers and mTOR signaling pathway-related proteins in the hippocampus and SH-SY5Y cells. Centrifuged each hippocampal tissue after homogenization. Using RIPA lysis solution to extract proteins on ice. After SDS-PAGE electrophoresis, transferred the protein onto a PVDF membrane, blocked with 5% skim milk powder solution, and added a primary antibody at the dilution ratio of Beclin-1, LC3 II, p62, mTOR, p-mTOR, p70s6K, 4EBP1 1:1000, β-actin 1:10000. After blocking with 5% skimmed milk powder, added the primary antibody. The dilution ratio was Beclin-1, LC3 II, p62, mTOR, p-mTOR, p70s6K and 4EBP1 (1:1000), β-actin (1:10000), 4°C incubated overnight. Incubating secondary antibody (1:10,000) for 2 h, developed by ECL substrate chemiluminescence and exposure and development via Bio-Rad Chemiluminescence Imaging System. Using Image J software to determine the ratio of the gray value of the target proteins and GAPDH.

## 3 Results

The primary results are shown in Table 2.

**Table 2:** Primary result.

	Groups	Metrics	Primary results	Brief findings
*In vivo*	Control Model Donepezil LG-L LG-M LG-H	Memory and learning capacity	LGZG prolonged the escape latency and increased the platform-crossing times in mice	LGZG can enhance mice’s memory and learning capacity.
		Aβ plaque deposition	LGZG reduced hippocampal Aβ plaque deposition	LGZG can alleviate AD-characteristic pathology.
		Autolysosomes and mitochondria	LGZG increased the number of autolysosomes and normal mitochondria	LGZG can restore autophagic function in APP/PS1 mices.
		Beclin-1, LC3 II and p62 expression levels	LGZG increased Beclin-1 and LC3 II expression and decreased p62 expression.	LGZG can activate autophagy in APP/PS1 mices.
		mTOR pathway-associated protein expression	LGZG reduced p-mTOR and p70S6K protein levels while elevating 4EBP1.	LGZG can inhibit mTOR signaling pathway in APP/PS1 mices.
*In vitro*	Control Model Rapa LG-L LG-L + 3-MA LG-M LG-M + 3-MA LG-H LG-H + 3-MA	Total cellular activity	The cytoprotective effect of LGZG-Medicated Serum (LMS) was partially diminished by 3-MA.	The neuroprotective effect of LMS is partially mediated through the mTOR signaling pathway.
		Autophagic vesicles	LMS increased autophagic vacuoles’ number	LMS may play a cytoprotective role by promoting autophagic vacuole formation.
		Beclin-1, LC3 II, and p62 expression levels	LMS enhanced Beclin-1 and LC3 II expressions while reduced p62 expression, and this phenomenon was partially attenuated by 3-MA.	LMS can promote autophagy in SH-SY5Y cells induced by Aβ_25-35_, and 3-MA can partially reduce this effect.
		Expression of the mTOR pathway related proteins	LMS decreased the expression levels of p-mTOR and p70S6K, while 4EBP1 expression showed an opposite trend.	LMS can modulate the mTOR pathway in Aβ_25-35_-induced SH-SY5Y cells, and 3-MA can partially reduce this effect.

### 3.1 Six components of LGZG were identified by HPLC

**Figure 1:**
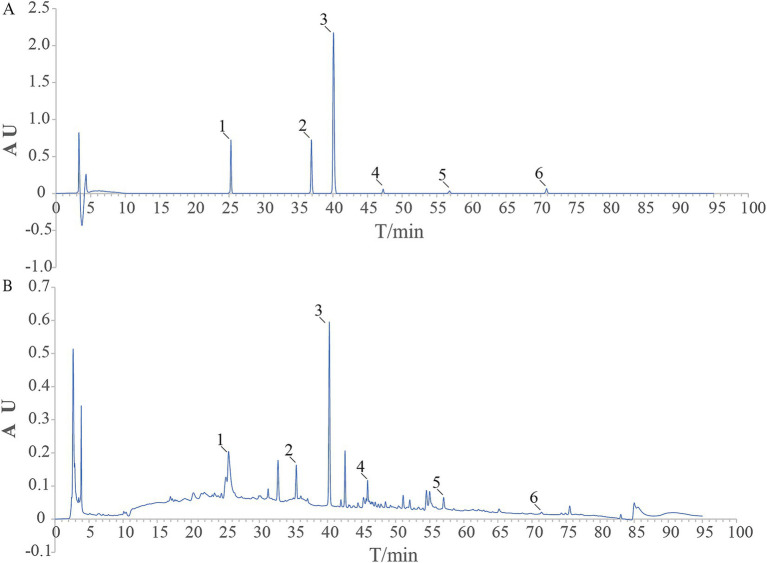
HPLC chart of the reference substance **(A)** and LGZG sample **(B)** 1. Liquiritin; 2. Cinnamic acid; 3. Cinnamaldehyde; 4. Atractylenolide III; 5. Glycyrrhizic acid; 6. Pachymic acid.

Using HPLC to make a determination of components of LGZG decoction. The HPLC fingerprint of LGZG decoction is shown in Figure 1B, and the control fingerprint is shown in Figure 1A. After data processing, a total of 6 chromatographic peaks were calibrated. The six components in the LGZG decoction and their corresponding peak times were Liquiritin, Cinnamic acid, Cinnamaldehyde, Atractylenolide III, Glycyrrhizic acid, Pachymic acid, which was consistent with mixed standards. It showed that the LGZG used in this experiment contains the six components mentioned above.

### 3.2 LGZG ameliorated memory impairment and alleviated Aβ pathology in APP/PS1 mice

**Figure 2:**
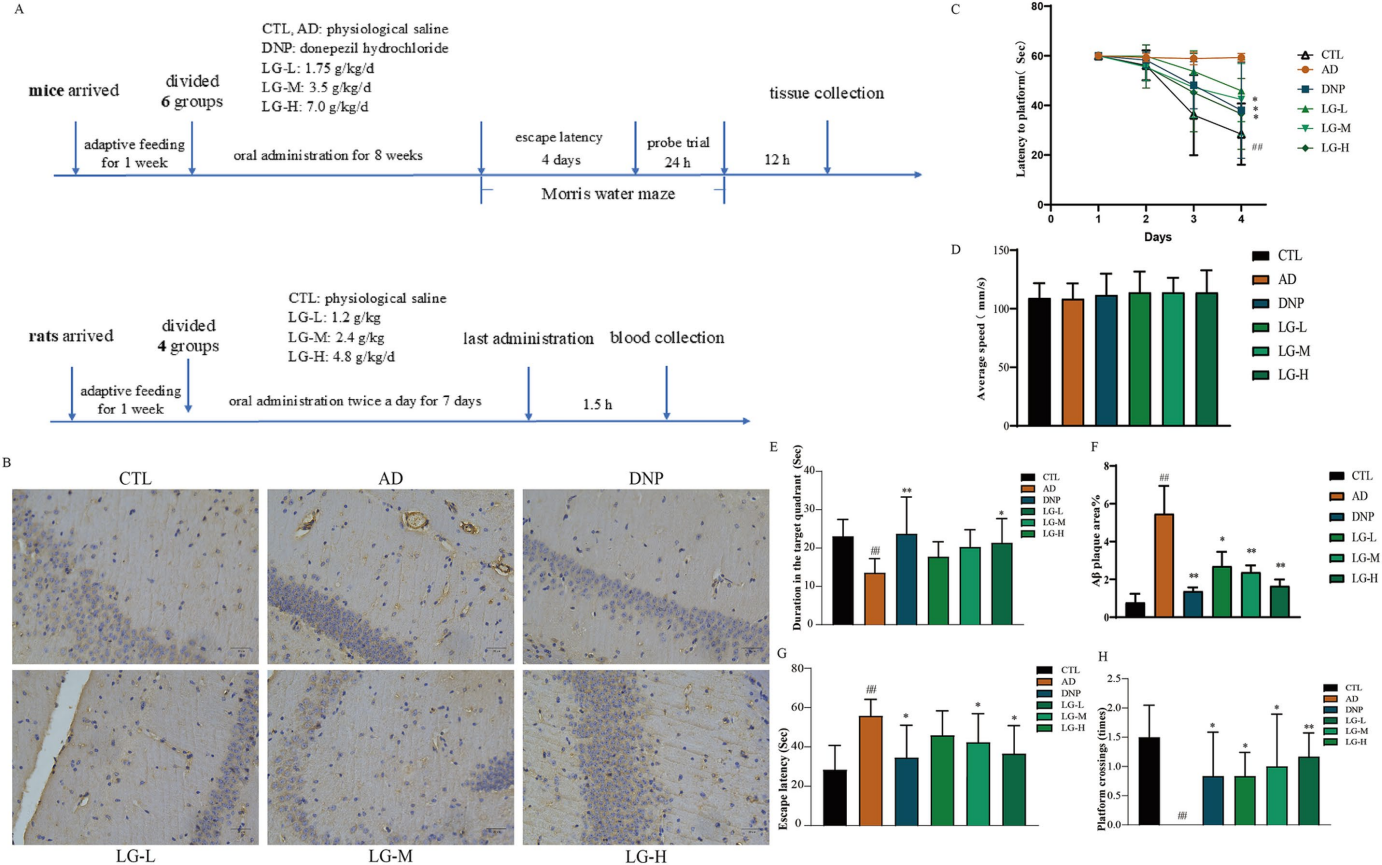
LGZG ameliorated memory impairment and alleviated Aβ pathology in APP/PS1 mice. **(A)** The dosing schema; **(B)** Immunohistochemistry of hippocampal slices (20 μm); **(C)** Escape latency in the MWM in 4 days; **(D)** Swimming speed in MWM; **(E)** Duration in the target quadrant; **(F)** Histogram of percentage of Aβ plaque area; **(G)** Escape latency; **(H)** Crossing times. Data are expressed as mean ± SEM, *n* = 5, ^##^ *p* < 0.01 with the control (CTL) group, *p < 0.05 and **p < 0.01 compared with the disease (AD) group.

The dosing schema is shown in Figure 2A. As presented in Figure 2C, except for the disease group, the escape latency of the other groups tended to decrease after training. As shown in Figure 2G, the Mean Morris Water Maze escape latency on day 4 was significantly longer in the disease group of mice than in the control group, indicating that cognitive function was impaired in APP/PS1 mice. In addition, LGZG (LG-M, *p* < 0.05; LG-H, *p* < 0.05 vs. the AD) and donepezil (*p* < 0.05 vs. the AD) treatment significantly improved the extended escape latency. In the LG-L group, the escape latency was also decreased, but not statistically significant. In the spatial probe test of the Morris Water Maze, the previous platform was removed, and the mice searched for the platform based on previous training memories, the larger the number of times of crossing the platform and the longer the time spent at the location of the original platform indicated better memory function. As illustrated in Figure 2H, the disease group had significantly fewer platform crossing times in the probe test than the control group (*p* < 0.01). Platform crossing times were increased in the intervention group compared to the disease group, and the results were statistically significant in the LG-M group, LG-H group, and the donepezil group (*p* < 0.05 vs. the AD). The duration results in the target quadrant of Morris Water Maze are shown in Figure 2E. The control group stayed in the quadrant with the platform longer than the disease group (*p* < 0.01). We also found a significant increase in the LG-H group (*p* < 0.05 vs. the AD) and the donepezil group (*p* < 0.01 vs. the AD) concerning duration in the target quadrant. The above results are based on the fact that there is no significant difference in swimming speed among the groups (Figure 2D, *p* > 0.05). These results indicated that LGZG can enhance the memory and learning capacity of APP/PS1 transgenic mice in the Morris Water Maze test. In addition, findings from this study showed nearly no Aβ positivity and Aβ deposition in the hippocampal slices of the control group (Figure 2B), while the disease group exhibited marked Aβ deposition. Hippocampal regions of the donepezil group (*p* < 0.01 vs. the AD), LG-H group (*p* < 0.01 vs. the AD), LG-M group (*p* < 0.01 vs. the AD), and LG-L group (*p* < 0.05 vs. the AD) displayed significantly less Aβ positive staining than the control group (Figure 2F). These data demonstrate that LGZG reduces hippocampal Aβ plaque deposition in APP/PS1 mice.

### 3.3 LGZG improved the hippocampal ultrastructure and regulated the expression level of autophagy related proteins in APP/PS1 mice

**Figure 3:**
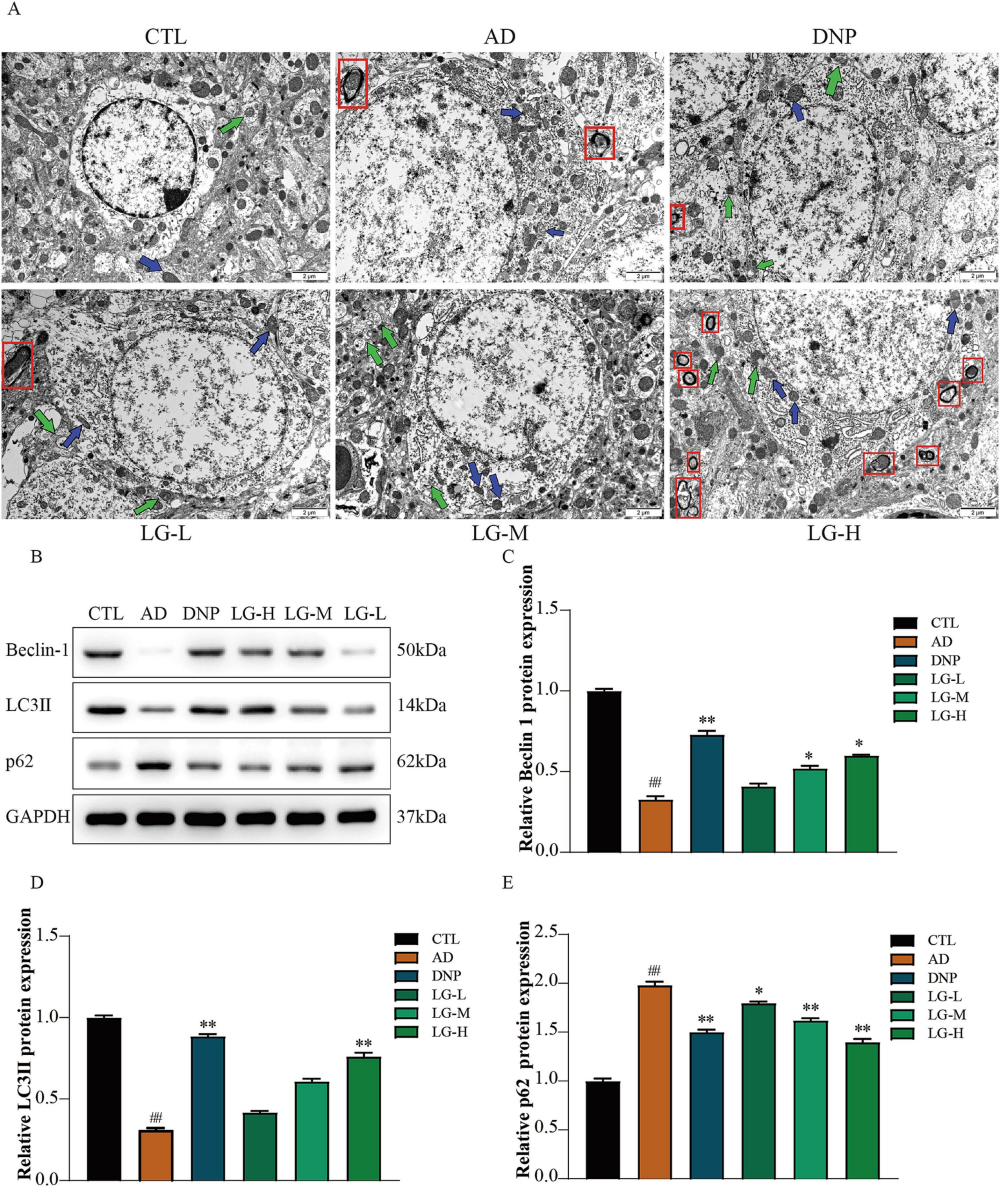
LGZG improved the hippocampal ultrastructure and regulated the expression level of autophagy related proteins in APP/PS1 mice. **(A)** Ultrastructural examination of the hippocampus (10,000x). **(B)** Western blot bands; **(C)** Beclin-1; **(D)** LC3 II; **(E)** p62. Data are expressed as mean ± SEM, n = 5, ^##^ *p* < 0.01 with the control (CTL) group, *p < 0.05 and **p < 0.01 compared with the disease (AD) group.

We then performed the ultrastructural examination of the hippocampus (Figure 3A). In the control group, the nuclei and organelles were intact, and mitochondrial cristae were transparent. In the disease group, the mitochondria swelled, the mitochondrial membrane partially disappeared, and the mitochondrial ridge fused and even disappeared. The neuronal myelin sheath structure was loose and appeared to dissolve. No autophagy-lysosome was utterly degraded, suggesting a possible impairment of autophagy in APP/PS1 mice. The number of autolysosomes and normal mitochondria increased in the LG-L, LG-M, and LG-H groups, as well as in the donepezil group. LGZG was also observed can activate autophagy in APP/PS1 mice in the hippocampus by Western blot analysis (Figures 3B–E). There was a considerable increase of Beclin-1 and LC3 II expression in the disease group and a (*p* < 0.01 vs. CTL). The LG-H group exhibited a markable increase of Beclin-1 and LC3 II expression and decreased p62 expression compared to the disease group (*p* < 0.01). The LG-M group showed an increase in Beclin-1 expression (*p* < 0.01 vs. AD) and a decrease in p62 expression (*p* < 0.05 vs. AD), while the increase of LC3 II expression was not statistically significant (*p* > 0.05 vs. AD).

### 3.4 LGZG modulated the mTOR pathway in the hippocampus

**Figure 4:**
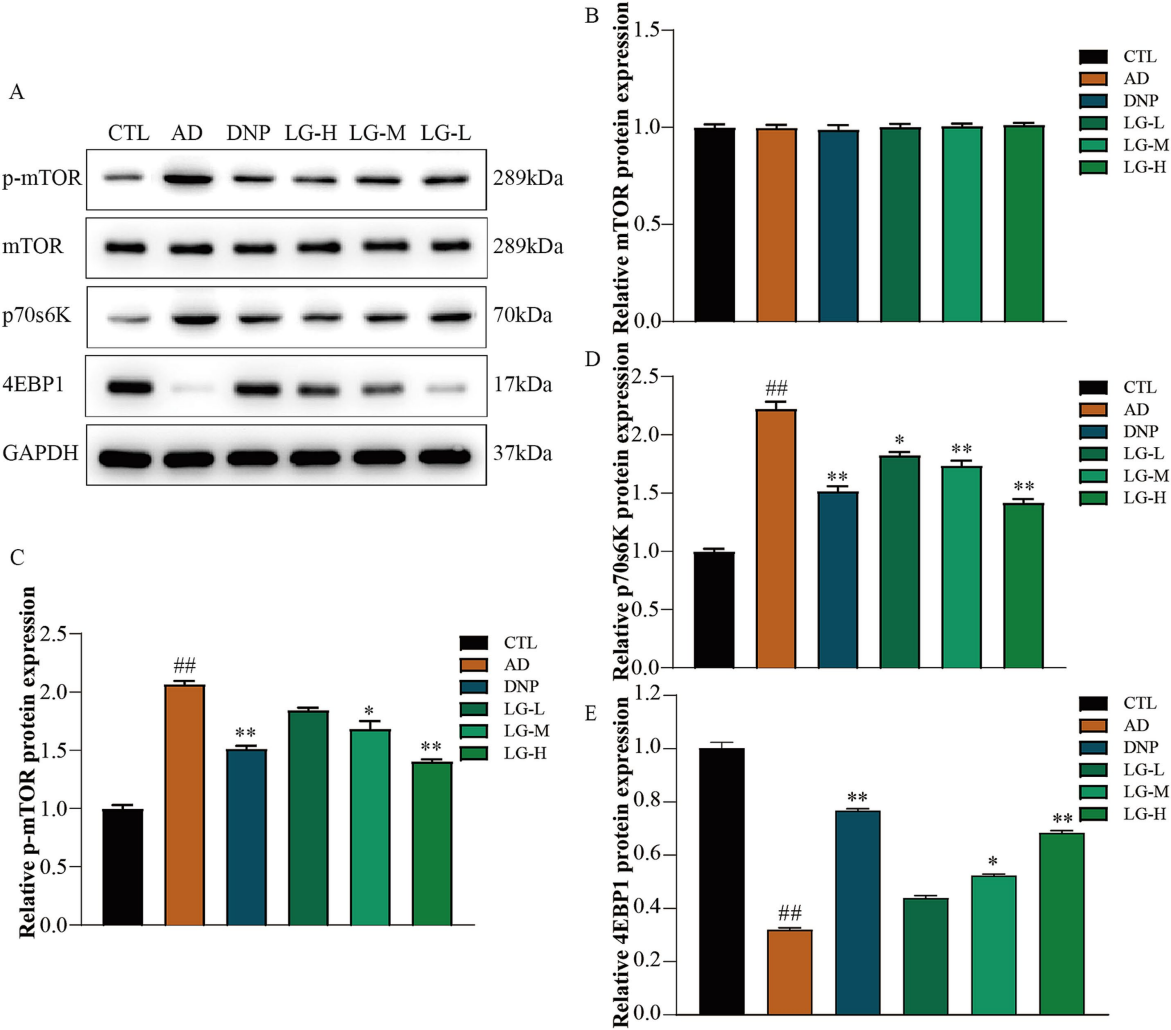
The mTOR, 4EBP1, and P70S6K expression in APP/PS1 mice intervened with LGZG. **(A)** Western blot bands; **(B)** mTOR; **(C)** p-mTOR; **(D)** p70s6K; **(E)** 4EBP1. Data are expressed as mean ± SEM, *n* = 5, ^##^ *p* < 0.01 with the control (CTL) group, *p < 0.05 and **p < 0.01 compared with the disease (AD) group.

Western blotting was used to evaluate the mTOR pathway-associated protein expression in APP/PS1 mice hippocampus. The results suggested that LGZG could significantly decrease the activation of the mTOR signaling pathway (Figure 4). The level of p-mTOR decreased in the LG-M group (*p* < 0.01 vs. AD) and the LG-H group (*p* < 0.05 vs. AD) as well as in the donepezil group (*p* < 0.01 vs. AD). p70S6K and 4EBP1 are the main downstream signaling molecules of mTOR. In our study, the change of trends p70S6K was consistent with the change of trends of p-mTOR in each group, while the changes in the trend of 4EBP1 were the opposite. This observation is following previous studies (Yang et al., 2019). These data indicated that LGZG inhibit mTOR signaling in APP/PS1 mice hippocampus.

### 3.5 Expressions of Beclin-1, LC3 II, and p62 at each time point in Aβ_25-35_-induced SH-SY5Y cells

**Figure 5:**
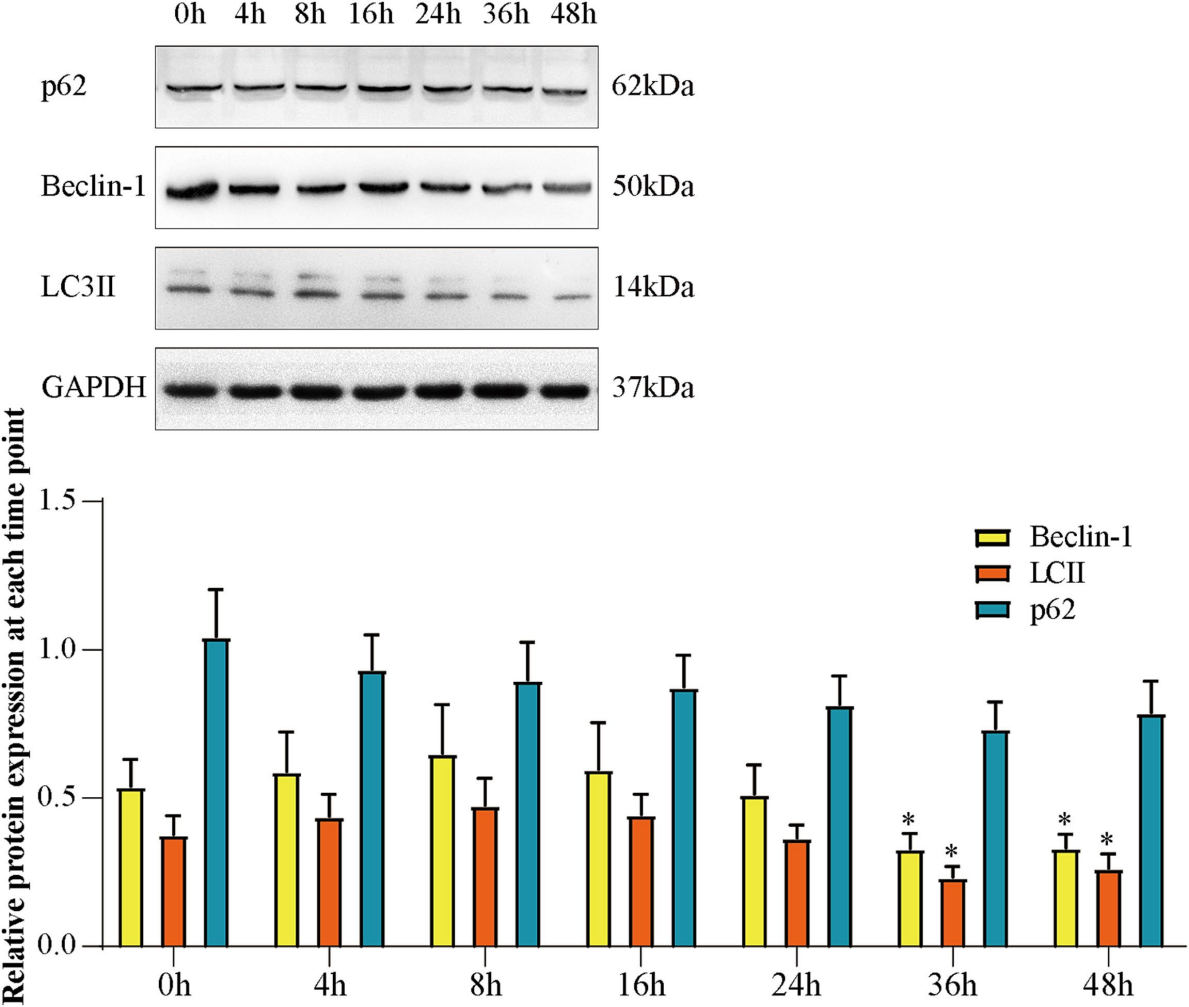
The expression of Beclin-1, LC3 II, and p62 at each time point of Aβ-induced SH-SY5Y cells. **(A)** Western blot bands; **(B)** expression of Beclin-1, LC3 II and p62. Data are expressed as mean ± SEM, *n* = 5, *p < 0.05 compared with the 0 h group.

We used Western blotting to analyze the expression of Beclin-1, LC3 II, and p62 in Aβ_25-35_-induced SH-SY5Y cells at different time points. As displayed in Figure 5, the expression of Beclin-1 and LC3 II increased and the expression of p62 decreased in the first 8 h, which demonstrated that autophagy was activated. The expression of Beclin-1 and LC3 II were significantly decreased at 36 h and the expression of p62 increased compared to 0 h (*p* < 0.05). This demonstrated that autophagy was inhibited. These results proved impaired autophagy occured at 36 h in Aβ_25-35_-induced SH-SY5Y cells.

### 3.6 LMS increased the activity of SH-SY5Y cells in an *in vitro* autophagy injury model and improved the autophagy status

**Figure 6:**
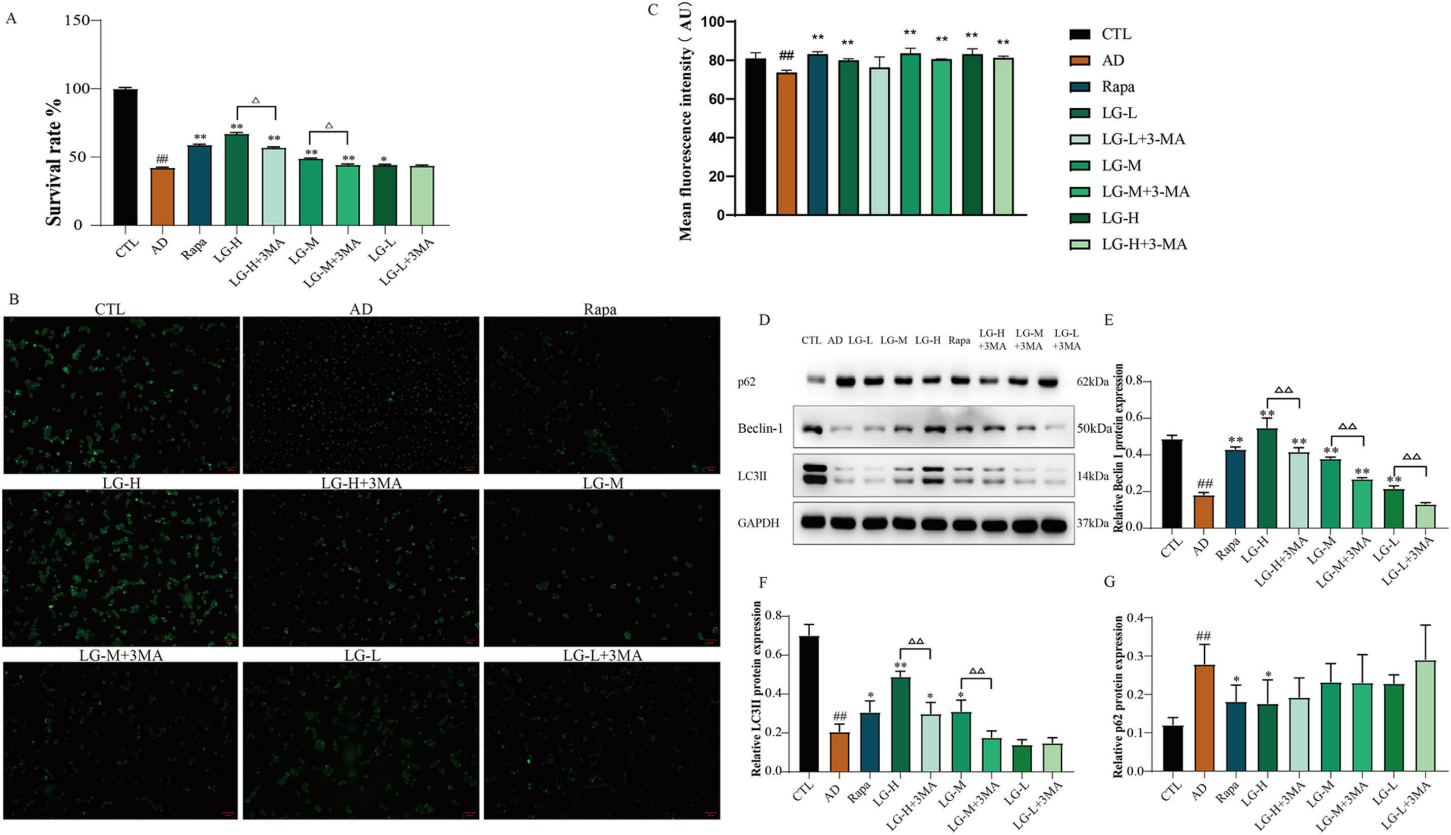
LMS increased the activity of SH-SY5Y cells in an *in vitro* autophagy injury model and improved the autophagy status. **(A)** Effects of LMS on the cellular activity of autophagy injury model. Data are expressed as mean ± SEM, n = 5, ^##^ *p* < 0.01 compared with the control (CTL) group, ^△^*p* < 0.05, ^△△^*p* < 0.01, *p < 0.05, and **p < 0.01 compared with the disease (AD) group. **(B)** MDC staining (50 μm). **(C)** Mean fluorescence intensity of MDC. n = 5; ^##^ *p* < 0.01 vs. CTL, * *p* < 0.05, ** *p* < 0.01 vs. AD. (CTL, 10% drug-free serum; AD, 20 μM Aβ_25-35_ + 10% drug-free serum; LG-L, 20 μM Aβ_25-35_ + 10% 1.2 g/kg LMS; LG-L + 3-MA, 20 μM Aβ_25-35_ + 10% 1.2 g/kg LMS + 5 mM 3-MA; LG-M, 20 μM Aβ_25-35_ + 10% 2.4 g/kg LMS; LG-M + 3-MA, 20 μM Aβ_25-35_ + 10% 2.4 g/kg LMS + 5 mM 3-MA; LG-H, 20 μM Aβ_25-35_ + 10% 4.8 g/kg LMS; LG-H + 3-MA, 20 μM Aβ_25-35_ + 10% 4.8 g/kg LMS + 5 mM 3-MA; Rapa, 20 μM Aβ_25-35_ + 100 nM Rapa +10% drug-free serum)**. (D-G)** The expression of autophagy-related proteins in SH-SY5Y cells **(D)** Western blot bands; **(E)** Beclin-1; **(F)** LC3 II; **(G)** p62. Data are expressed as mean ± SEM, *n* = 3, ^##^ *p* < 0.01 compared with the control (CTL) group, ^△^*p* < 0.05, ^△△^*p* < 0.01, *p < 0.05, and **p < 0.01 compared with the disease (AD) group. (CTL, 10% drug-free serum; AD, 20 μM Aβ_25-35_ + 10% drug-free serum; LG-L, 20 μM Aβ_25-35_ + 10% 1.2 g/kg LMS; LG-L + 3-MA, 20 μM Aβ_25-35_ + 10% 1.2 g/kg LMS + 5 mM 3-MA; LG-M, 20 μM Aβ_25-35_ + 10% 2.4 g/kg LMS; LG-M + 3-MA, 20 μM Aβ_25-35_ + 10% 2.4 g/kg LMS + 5 mM 3-MA; LG-H, 20 μM Aβ_25-35_ + 10% 4.8 g/kg LMS; LG-H + 3-MA, 20 μM Aβ_25-35_ + 10% 4.8 g/kg LMS + 5 mM 3-MA; Rapa, 20 μM Aβ_25-35_ + 100 nM Rapa +10% drug-free serum).

The total cellular activity was detected by MTT assay (Figure 6A). There was a decrease in cell activity in the disease group (*p* < 0.01 vs. CTL), and the cell activity significantly increased in the Rapa group (*p* < 0.01 vs. AD), suggesting that inhibition of mTOR signaling pathway could protect Aβ_25-35_-induced SH-SY5Y cells. Other groups except the LG-L + 3-MA group showed an increase in cellular activity compared with the control group (LG-L, *p* < 0.05. LG-M, LG-M + 3-MA, LG-H, LG-H + 3-MA, *p* < 0.01). In addition, LG-M + 3-MA (*p* < 0.01 vs. LG-M) and LG-H + 3-MA (*p* < 0.01 vs. LG-H) decreased in cellular activity, suggesting the cytoprotective effect of LMS could be partially reduced but not abolished by 3-MA. Monodansylcadaverine (MDC) is a fluorescent compound which specifically labels autophagic vesicles because the acidic environment within these vesicles triggers MDC to emit fluorescence (Biederbick et al., 1995). By observing the results of MDC staining (Figure 6B) and the detection of average fluorescence intensity (Figure 6C), we found that the disease group’s moderate fluorescence intensity was significantly lower than the control group (*p* < 0.01), indicating the absence of autophagy vesicles in the disease group. After drug intervention, the mean fluorescence intensity in all groups except the LG-L + 3-MA group significantly increased (*p* < 0.01 vs. CTL). The results were compatible with the MTT analysis, suggesting that LMS may play a cytoprotective role by promoting autophagic vacuole formation. We further examined autophagy associated proteins in the cells. As shown in Figures 6D–G, the expression of LC3 II and Beclin-1 were markedly decreased in the disease group and the expression of p62 was increased compared to the control group (*p* < 0.01 vs. CTL), suggesting that autophagy was impaired in the disease group. The expression of Beclin-1 increased in LG-L, LG-M, LG-M + 3-MA, LG-H, and LG-H + 3-MA groups, as well as in Rapa group (*p* < 0.01 vs. AD), and declined in LG-L + 3-MA (*p* < 0.01 vs. LG-L), LG-M + 3-MA (*p* < 0.01 vs. LG-M) and LG-H + 3-MA groups (*p* < 0.01 vs. LG-H). The expression of LC3 II increased in Rapa (*p* < 0.05 vs. AD), LG-H (*p* < 0.01 vs. AD), LG-H + 3-MA (*p* < 0.05 vs. AD) and LG-M (*p* < 0.05 vs. AD) groups, and decreased in LG-H + 3-MA (*p* < 0.01 vs. LG-H) and LG-M + 3-MA (*p* < 0.01 vs. LG-M) groups. The Rapa and LG-H groups showed a reduction in the expression of p62 (*p* < 0.05 vs. AD); other administered groups decreased the p62 expression, but these changes were statistically insignificant. These results suggested that LMS can promote autophagy in SH-SY5Y cells induced by Aβ_25-35_, and this effect can be partially reduced by 3-MA.

### 3.7 LMS modulated the expression of the mTOR pathway in Aβ_25-35_-induced SH-SY5Y cells

**Figure 7:**
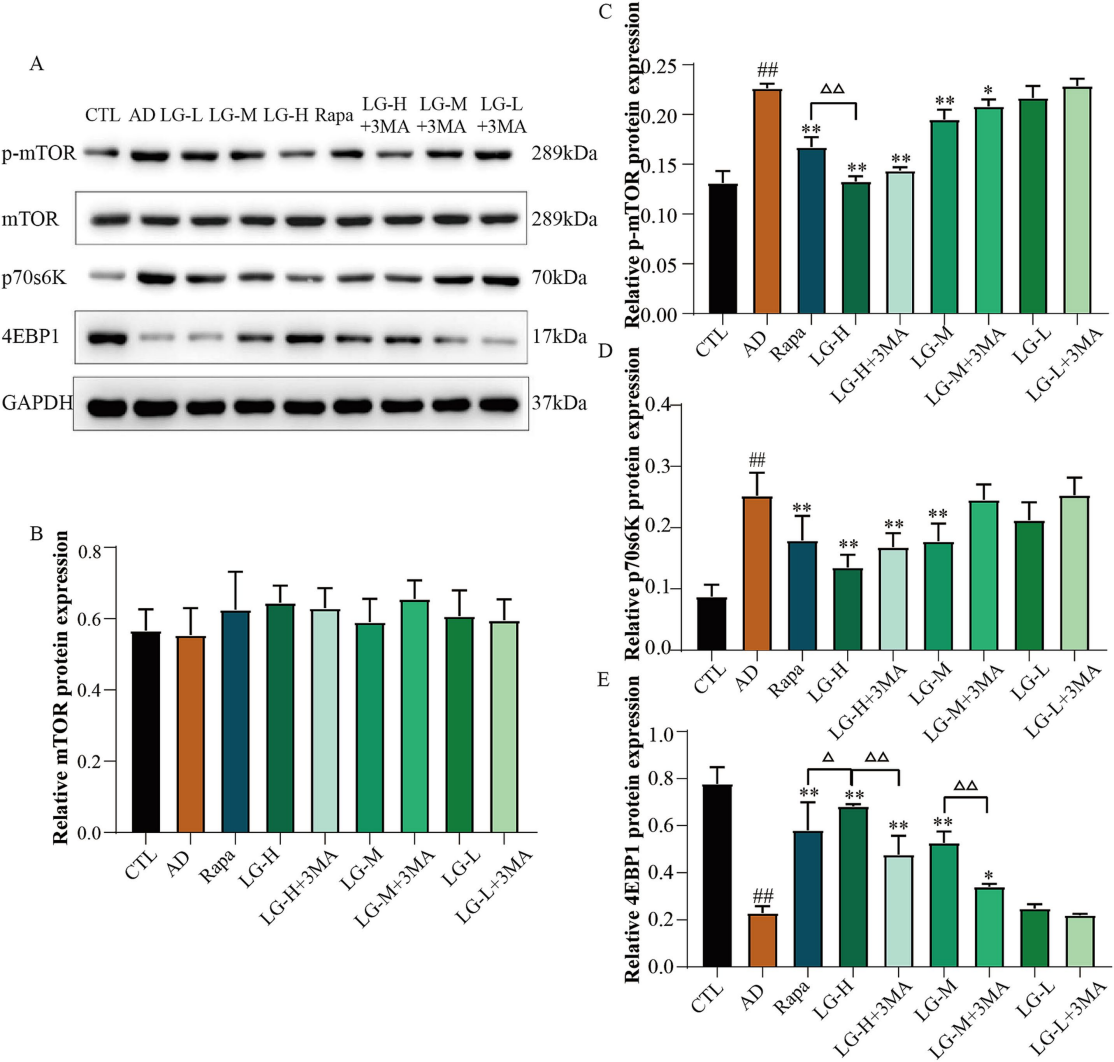
The expression of mTOR, 4EBP1, and P70S6K in APP/PS1 mice intervened with LGZG. **(A)** Western blot bands; **(B)** mTOR; **(C)** p-mTOR; **(D)** P70S6K; **(E)** 4EBP1. Data are expressed as mean ± SEM, *n* = 3, ^##^ *p* < 0.01 compared with the control (CTL) group, ^△^*p* < 0.05, ^△△^*p* < 0.01, *p < 0.05, and **p < 0.01 compared with the disease (AD) group. (CTL, 10% drug-free serum; AD, 20 μM Aβ_25-35_ + 10% drug-free serum; LG-L, 20 μM Aβ_25-35_ + 10% 1.2 g/kg LMS; LG-L + 3-MA, 20 μM Aβ_25-35_ + 10% 1.2 g/kg LMS + 5 mM 3-MA; LG-M, 20 μM Aβ_25-35_ + 10% 2.4 g/kg LMS; LG-M + 3-MA, 20 μM Aβ_25-35_ + 10% 2.4 g/kg LMS + 5 mM 3-MA; LG-H, 20 μM Aβ_25-35_ + 10% 4.8 g/kg LMS; LG-H + 3-MA, 20 μM Aβ_25-35_ + 10% 4.8 g/kg LMS + 5 mM 3-MA; Rapa, 20 μM Aβ_25-35_ + 100 nM Rapa +10% drug-free serum).

Western blot was used to analysis the expression of the mTOR pathway related proteins (Figure 7). The levels of p-mTOR and p70S6K were remarkably elevated in a condition where the expression of mTOR was not significantly different among all groups (*p* > 0.05). The expression of 4EBP1 was significantly declined in the disease group (*p* < 0.01 vs. CTL), while Rapa significantly inverted this phenomenon (*p* < 0.01 vs. AD). p-mTOR levels were increased in the LG-H (*p* < 0.01 vs. AD), LG-H + 3-MA (*p* < 0.01 vs. AD), LG-M (*p* < 0.01 vs. AD), and LG-M + 3-MA (*p* < 0.05 vs. AD) groups compared with the disease group, and 4EBP1 expression showed an opposite trend. In addition, LG-H offered more substantial regulatory effects on the p-mTOR and 4EBP1 than Rapa (*p* < 0.01 vs. Rapa), and this phenomenon could be weakened by 3-MA (*p* > 0.01 LG-H + 3-MA vs. Rapa). These results suggested LMS can modulate the mTOR pathway in Aβ_25-35_-induced SH-SY5Y cells, and 3-MA can partially reduce this effect.

## 4 Discussion

Autophagy is an essential pathway of Aβ clearance in the brain. During the development of AD, autophagy will be upregulated by the induction of Aβ. Still, with the continual increase of Aβ deposition, autophagy will eventually be inhibited and lose the ability to maintain its clearance function. Dysfunctional autophagy exacerbates intracellular Aβ accumulation and extracellular Aβ plaque formation (Esteves et al., 2019). Our results suggested that APP/PS1 mice exhibit significantly increased hippocampal Aβ deposition associated with behavioral deficits. Besides, the results showed that LGZG and donepezil treatment reduced the formation of Aβ plaques and repaired cognitive function in APP/PS1 transgenic mice.

Beclin-1 plays a vital role in the formation of autophagosomes, and Beclin-1 interacts with various cofactors to trigger the autophagy cascade reaction (Toton et al., 2014). Researches showed the level of Beclin-1 is depressed in the AD brain, and Beclin-1 gene defects can also lead to Aβ accumulation and neurodegeneration in mice. At the same time, activation of Beclin-1 can prevent neuron death and improve the clearance of toxic protein aggregates (Esteves et al., 2019). LC3 can bind to DLPE under the action of ATG4 and transform from LC3 I to active LC3 II, which is the key to the formation of autophagosomes, so LC3 II is considered to be the landmark protein of autophagy (Heras-Sandoval et al., 2014). p62 is an essential protein of selective autophagy. It can label the substances which need to be degraded and degraded by autophagy along with autophagosomes, so p62 is negatively correlated with autophagy. Here, we found that autophagic flux was impaired, as evidenced by elevated levels of p62 and reduced LC3 II and Beclin-1 in the disease group mice, suggesting that both autophagosome formation and degradation were impaired in AD. Notably, the impaired autophagy status was repaired in this study by treated with different concentration LMS. This study demonstrated that LGZG treatment upregulated the LC3 II and Beclin-1 expression and downregulated p62 levels.

mTOR plays a vital role in AD autophagy deficiency. mTOR inhibits autophagy by reducing the activity of ubiquitin-like kinase (ULK) (Glick et al., 2010). Many clinical and animal researches have revealed that the disorder of the mTOR signaling pathway is related to autophagy disorder in the AD patients’ brain (Sun et al., 2014; Tramutola et al., 2015; Zhang et al., 2017). 4EBP1 and p70s6K are the primary downstream targets of the mTOR pathway. The overactivation of the mTOR signal pathway decreases 4EBP1 and increases p70s6K levels in APP/PS1 mice, and an increased p70s6K expression was reported to exacerbate the hyperphosphorylation of tau and the deposition of Aβ (Pei et al., 2008). We explored the potential mechanism by detecting mTOR and its downstream proteins, p70S6K, and 4EBP1 phosphorylation levels. Our study found that LGZG can inhibit mTOR hyperphosphorylation, reduce p70S6K activity, increase 4EBP1 protein levels, and promote autophagy.

In this *in vitro* assay, we confirmed the different time points of 20 μM Aβ_25-35_-induced SH-SY5Y cells appear to have impaired autophagy at 36 h. Based on this, we established the autophagy injury cell model. Our results suggested that LGZG-Medicated serum can increase the LC3 II and Beclin-1 expression, reduce p62 levels, and inhibit the activity of mTOR. These results were consistent with those observed *in vivo*. Furthermore, we found these effects can be partially reduced by 3-MA.

This study found that LGZG administration ameliorates cognitive impairment in APP/PS1 transgenic mice. Aβ plaques in the brain are a key pathological feature of AD, the immunohistochemistry results demonstrated that LGZG treatment reduces the formation of Aβ deposition. The Western blotting assay results indicated that LGZG can upregulate LC3 II and Beclin-1, downregulate p62, and suppress aberrant mTOR pathway phosphorylation *in vivo* and *in vitro*. These effects could be partially reduced by 3-MA, which is a classic III phosphatidylinositol 3-kinase (PI3K) inhibitor. Therefore, we assumed that LGZG can activate autophagy by inhibiting the mTOR pathway, thereby improving cognitive function and promoting Aβ clearance, and the regulatory effect of LGZG on mTOR is only partially dependent on the PI3K/mTOR signaling pathway.

Donepezil is a cholinesterase inhibitor. By inhibiting cholinesterase activity, it slows down the decomposition of acetylcholine in the synaptic cleft of neurons, thereby increasing the content of acetylcholine and improving the clinical symptoms of AD patients (Zhang and Gordon, 2018). Donepezil has demonstrated a range of effects, including protecting against amyloid β, ischemia and glutamate toxicity; slowing of progression of hippocampal atrophy (Kim et al., 2017). The therapeutic effect of LGZG is more focused on Aβ clearance. Our team has previously confirmed that LGZG can protect the normal physiological functions of the blood–brain barrier and glymphatic system and promote Aβ clearance. In addition, LGZG may also have the potential to interfere with meningeal lymphatic drainage in AD, and the relevant research is ongoing.

There are several limitations to this study. First, liquid chromatography-mass spectrometry (LC–MS) technology can be used to further detect the active ingredients in LGZG containing serum and clarify its pharmacological substance basis. Second, the conclusion that LGZG can regulate mTOR pathway lacks further experimental verification, we need pay more efforts to conduct more in-depth and comprehensive experiments to fill the current research. Finally, we cannot rule out the possibility of LGZG regulating autophagy by non-mTOR pathways. Because we added PI3K inhibitors, the LGZG+3MA group also showed enhanced autophagy, indicating that the regulation of autophagy by LGZG may also depend on other pathways. It is necessary to explore this in future research.

## 5 Conclusion

In summary, this study investigated the autophagy regulatory mechanisms of LGZG in AD models. Our findings showed that LGZG can significantly modulate the expression of autophagy-related proteins by inhibiting the mTOR signaling pathway, reducing p-mTOR and p70S6K levels while increasing 4EBP1, suggesting its potential therapeutic effect on AD. The effects above were partially attenuated by 3-MA, the autophagy inhibitor. These results suggested that LGZG exerted neuroprotective effects through the autophagy pathway, providing a potential new approach for the treatment of AD.

## Data Availability

The original contributions presented in the study are included in the article/supplementary material, further inquiries can be directed to the corresponding author.
